# Nonenzymatic polymerase-like template-directed synthesis of acyclic l-threoninol nucleic acid

**DOI:** 10.1038/s41467-021-21128-0

**Published:** 2021-02-05

**Authors:** Keiji Murayama, Hikari Okita, Takumi Kuriki, Hiroyuki Asanuma

**Affiliations:** Graduate School of Engineering, Nagoya University, Furo-cho, Chikusa-ku, Nagoya, 464-8603 Japan

**Keywords:** DNA, Nucleic acids, Origin of life

## Abstract

Evolution of xeno nucleic acid (XNA) world essentially requires template-directed synthesis of XNA polymers. In this study, we demonstrate template-directed synthesis of an acyclic XNA, *acyclic* l-threoninol nucleic acid (l-*a*TNA), via chemical ligation mediated by *N*-cyanoimidazole. The ligation of an l-*a*TNA fragment on an l-*a*TNA template is significantly faster and occurs in considerably higher yield than DNA ligation. Both l-*a*TNA ligation on a DNA template and DNA ligation on an l-*a*TNA template are also observed. High efficiency ligation of trimer l-*a*TNA fragments to a template-bound primer is achieved. Furthermore, a pseudo primer extension reaction is demonstrated using a pool of random l-*a*TNA trimers as substrates. To the best of our knowledge, this is the first example of polymerase-like primer extension of XNA with all four nucleobases, generating phosphodiester bonding without any special modification. This technique paves the way for a genetic system of the l-*a*TNA world.

## Abstract (web summary)

A world preceding the prebiotic RNA-world may have been based on xeno nucleic acids (XNAs), but their replication likely did not require enzymes. Here, the authors demonstrate template-directed non-enzymatic synthesis of an XNA, acyclic l-threoninol nucleic acid, via chemical ligation mediated by N-cyanoimidazole, and achieve a pseudo-primer extension of this XNA with all four nucleobases.

## Introduction

The RNA world is widely thought to be a stage in the origin of life^1–4^. At this stage, RNA self-replication must have occurred from simple inorganic and organic molecules that existed on ancient Earth. To provide support for the RNA world hypothesis, nonenzymatic self-replication of oligoribonucleotide via efficient chemical ligation of monomer units or short fragments under aqueous conditions in the presence of metal ions and simple activators has been investigated. Orgel and co-workers have primarily reported that activated nucleotide monomers bearing a leaving group on phosphate can be polymerized on a template oligonucleotide to produce a complementary strand^5,6^. Richert’s group Hagenbuch et al. and Deck et al. utilized azabenzotriazolide as an effective leaving group and a helper strand to achieve effective primer extension^7,8^. Orgel and Szostak introduced an amino group into 2′- and 3′-positions^9^, which enabled effective RNA copying due to acceleration of the ligation reaction^10,11^. These investigations indicated that prebiotic conditions may have allowed ribo-oligonucleotide elongation on a template strand in the absence of a protein enzyme.

**Fig. 1:**
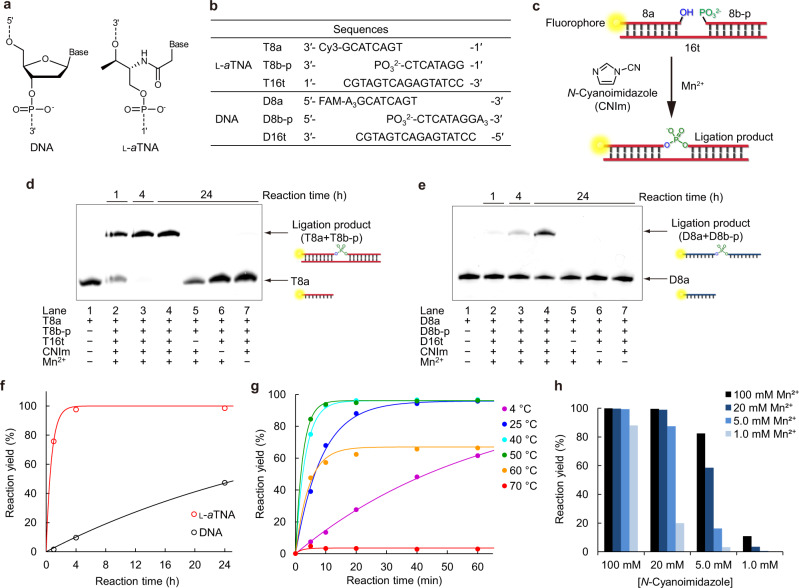
Chemical ligation of l-*a*TNA and DNA. **a** Chemical structures of DNA and l-*a*TNA. **b** Sequences of 8-mer fragments and 16-mer templates. **c** Schematic illustration of the chemical ligation reaction. Denaturing PAGE analysis of **d** l-*a*TNA ligation reaction and **e** DNA ligation reaction. Lane 1, X8a as a marker; lane 2, reaction products after 1 h; lane 3, reaction products after 4 h; lane 4, reaction products after 24 h; lane 5, products after reaction in the absence of template; lane 6, products after reaction in the absence of CNIm; and lane 7, products after reaction in the absence of Mn^2+^. Conditions: [X8a]  =  0.9 μM, [X8b-p] = 1.1 μM, [X16t] = 0 or 1.0 μM, [NaCl] = 100 mM, [MnCl_2_] = 0 or 20 mM, [CNIm] = 0 or 20 mM, 4 °C. Source data are provided as a Source Data file. **f** Chemical ligation yield of l-*a*TNA (red circles) and DNA (black circles) after indicated times of reaction. **g** Chemical ligation yields of l-*a*TNA at indicated temperatures. Conditions: [oligomers] = 1.0 μM each, [NaCl] = 100 mM, [MnCl_2_] = 20 mM, [CNIm] = 20 mM. Source data are provided as a Source Data file. **h** Ligation yields of l-*a*TNA at indicated concentrations of CNIm and Mn^2+^. Conditions: [T8a] = 0.9 μM, [T8b-p] = 1.1 μM, [T16t] = 1.0 μM, [NaCl] = 100 mM 25 °C, 30 min. Source data are provided as a Source Data file.

Why nature chose ribofuranosyl nucleic acids rather than some other family of molecular structure as the carrier of genetic material^12^ has prompted chemists to synthesize various xeno nucleic acids (XNAs) composed of unnatural scaffolds^13,14^. Cyclic XNAs such as hexitol nucleic acid^15^, cyclohexenyl nucleic acid^16^, tricyclo-DNA^17^, arabinonucleic acid^18^, and (3′,2′)-α-L-threose nucleic acid (TNA)^12,19^ form homoduplexes and cross-pair with DNA and/or RNA. Due to the structural similarities to DNA and RNA, cyclic XNAs can be polymerized on a template using native and engineered polymerases, and functional XNAs that act as ribozymes and aptamers have been identified using in vitro selection^20–26^. TNA has particularly attracted attention because it is chemically simpler than RNA and may have existed prior to RNA^19,27–30^. Interestingly, a ring structure is not necessary for stable duplex formation. Several XNAs composed of acyclic structures have been synthesized^31–38^. An example is glycerol nucleic acid (GNA), designed by the Zhang et al.^34^. Although GNA does not have a ring structure, it forms very stable homoduplex and can cross-pair with RNA, depending on the sequence. As other examples, we have reported serinol nucleic acid^39^ and *acyclic* l-threoninol nucleic acid (l-*a*TNA; Fig. 1a)^40,41^, which can cross-hybridize with DNA and RNA and has high nuclease durability, promising application to wide variety of situations such as living cell system^42–44^. Not only our group but Kumar, Gothelf, et al. Kumar et al., Kumar and Gothelf, and Kumar et al. have also reported unique properties of l-*a*TNA^45–47^.

If template-directed synthesis of an XNA could occur in the absence of enzymes, the XNA world would become a more likely candidate for a pre-RNA world. Szostak’s group has used chemical ligation to polymerize 2′-amino-modified GNA^48^. Liu and co-workers reported that peptide nucleic acid (PNA) aldehydes could be chemically ligated on a DNA template, generating a complementary PNA strand^49^. Inspired by these works, we focused on template-mediated chemical ligation of l-*a*TNA as the first step of achieving the acyclic XNA world, because (1) l-*a*TNA is synthetically and structurally simple, (2) l-*a*TNA can form very stable homoduplex in an antiparallel manner, and (3) l-*a*TNA with clockwise helicity can also form stable duplex with RNA as well as DNA. A condensing reagent *N*-cyanoimidazole (CNIm) can connect a phosphate with a neighboring hydroxyl group to generate a phosphodiester bond, the same type of bond formed with natural enzymatic ligation^50,51^. For DNA, ligation of various types of structures were achieved using CNIm, due to relatively high reaction yields^52–56^. We therefore expected that CNIm could be used to ligate l-*a*TNA. If short fragments can be sequentially ligated on a template, selective and effective synthesis of complementary strands is possible as shown for other types of nucleic acids^11,49,57,58^.

Herein, we demonstrate template-directed chemical ligation of l-*a*TNA fragments, which is of interest from the viewpoint of the prebiotic world and from the standpoint of establishment of XNA-based artificial life. The chemical ligation of l-*a*TNA gives considerably higher yield and rate than DNA ligation. This effective ligation enables a pseudo-primer extension reaction using a pool of random l-*a*TNA trimers as substrates.

## Results and discussion

### Template-directed chemical ligation of l-*a*TNA in the presence of *N*-cyanoimidazole and divalent metal ion

We synthesized an 8-mer l-*a*TNA fragment with the fluorophore Cy3 conjugated at the 3′ terminus (T8a) and a fragment carrying a phosphate at the 3′ terminus (T8b-p). Both are complementary to a 16-mer l-*a*TNA template (T16t) (Fig. 1b). These three strands can form a duplex with single nick at the center. Upon addition of CNIm, we expected that the 1′-OH of T8a and the 3′-phosphate of T8b-p would be connected to generate a phosphodiester linkage (Fig. 1c). DNA oligonucleotides of almost the same sequences (D8a, D8b-p, and D16t) were prepared as a comparison. First, we evaluated ligation efficiency of the l-*a*TNA fragments upon addition of CNIm in the presence of Mn^2+^, conditions used in previous reports^50,54^. The ligation reaction was performed at 4 °C, a temperature at which both l-*a*TNA and DNA strands form duplexes. Products were analyzed by denaturing polyacrylamide gel electrophoresis (PAGE). Over time, the intensity of the band corresponding to T8a decreased and that of a band with slower mobility increased (Fig. 1d). The analysis of the reaction mixture by MALDI-TOF MS revealed that this new band contained the desired 16-mer ligation product of T8a and T8b-p (Calcd: 5879.6, Obs: 5877.3, Supplementary Fig. 1). This ligated product was not observed in the absence of T16t, Mn^2+^, or CNIm. The ligation reaction efficiency was high with 95% yield after 4 h. In contrast, the DNA ligation occurred in only 10% under the same conditions, and only 47% yield of DNA product was detected after 24 h (Fig. 1e, f). The observed rate constants (*k*_obs_) were estimated as 1.4 h^−1^ for l-*a*TNA and 0.027 h^−1^ for DNA. Thus, the l-*a*TNA ligation rate was about 50-fold higher than the DNA ligation rate.

Examination of ligation reaction yields over a range of temperatures revealed that the reaction rate was accelerated when the temperature was elevated to 50 °C (Fig. 1g and Supplementary Fig. 2): *k*_obs_ was 6.4, 16, and 21 h^−1^ at 25, 40, and 50 °C, respectively, resulting in almost 90% yield within 20 min at temperatures from 25 to 50 °C. Note that the reaction rate of l-*a*TNA octamer ligation was comparable to that of 3′-amino oligonucleotides with 2-methylimidazole-activated phosphate (about 7 h^−1^ at optimized conditions for decamer fragment)^11^, both are much faster than the rate of the chemical ligation to form a phosphodiester bond. In contrast, the l-*a*TNA octamer ligation reaction was suppressed at temperatures higher than the melting temperatures (*T*_m_) of the T8b-p/T16t duplex (59.5 °C), and thus no reaction was observed at 70 °C. This suggests that duplex formation between fragments and template is necessary for ligation, although CNIm may degrade at high temperatures. Examinations of the concentration dependences on CNIm and Mn^2+^ showed that 20 mM CNIm and 20 mM Mn^2+^ were sufficient for effective ligation (Fig. 1h and Supplementary Fig. 3). We also determined yields in Cd^2+^, Co^2+^, Ni^2+^, and Zn^2+^ and showed that each of these metal ions could substitute for Mn^2+^ (Supplementary Fig. 4). Thus, a wide variety of divalent metals induce ligation of l-*a*TNA.

### Position of phosphate and thermal stability of fragment duplex influence ligation rate

**Fig. 2:**
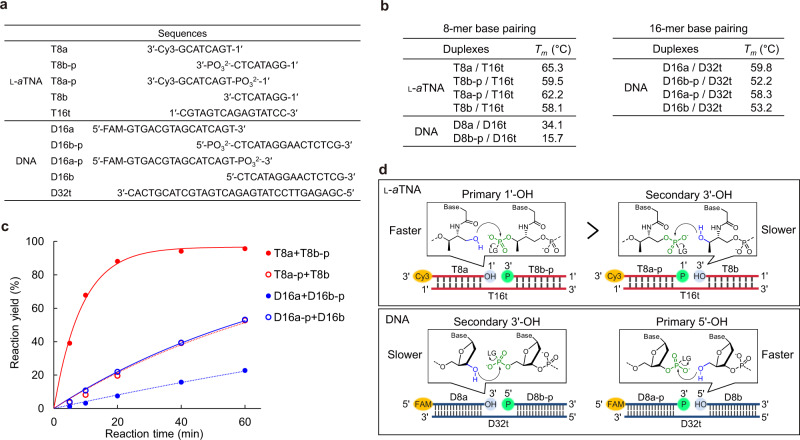
Evaluation of rate of chemical ligation. **a** Sequences of 8-mer fragments and 16-mer template of l-*a*TNA and 16-mer fragments and 32-mer template of DNA. **b** Melting temperatures of fragment/template duplexes. **c** Yield as a function of time for the chemical ligation reactions of l-*a*TNA (red circles) and of DNA (black circles) with either primary (closed circles) or secondary OH termini (open circles). Conditions: [oligomers] = 1.0 μM each, [NaCl] = 100 mM, [MnCl_2_] = 20 mM, [CNIm] = 20 mM, 25 °C. Source data are provided as a Source Data file. **d** Schematic illustration of chemical ligation focusing on condensation between OH and phosphate.

In order to examine the reasons for the remarkably high efficiency of l-*a*TNA ligation, we prepared and analyzed ligation reactions of l-*a*TNA and DNA of different sequences (Fig. 2a). T8a-p and T8b were designed to examine the effect of the position of the phosphate position on the ligation. Several 16-mer DNA fragments (D16a, D16b-p, D16a-p, and D16b) and a 32-mer template (D32t) were synthesized to prepare DNA duplexes with *T*_m_ values similar to that of 8-mer l-*a*TNA fragments and the 16-mer l-*a*TNA template (Fig. 2b and Supplementary Fig. 5). Interestingly, the position of terminal phosphate influenced the reaction rate. In the case of l-*a*TNA, the ligation was faster for the 1′-OH/3′-phosphate pair (T8a/T8b-p/T16t, *k*_obs_ = 6.4 h^−1^) than for the 1′-phosphate/3′-OH pair (T8a-p/T8b/T16t, *k*_obs_ = 0.61 h^−1^; Fig. 2c and Supplementary Fig. 6). For DNA, the ligation was faster for the 5′-OH/3′-phosphate pair (D16a-p/D16b/D32t, *k*_obs_ = 0.71 h^−1^) than for the 5′-phosphate/3′-OH pair (D16a/D16b-p/D32t, *k*_obs_ = 0.22 h^−1^, Fig. 2c and Supplementary Fig. 6). These differences could be partially explained by steric hindrance around the OH group, which acts as a nucleophile to attack the phosphorous atom of the phosphate activated by CNIm^50^. Both the 1′-OH on l-*a*TNA and the 5′-OH on DNA are less bulky primary hydroxyl groups than are the 3′-OH groups, which are bulky secondary hydroxyl groups (Fig. 2d). The same trend was reported for the chemical ligation of DNA using BrCN^59^ and EDC^60^ with nick ligation between 3′-phosphate/5′-OH resulting in higher yields than ligation between the 5′-phosphate/3′-OH pair. Thus, the primary hydroxyl group is an important factor in reactivity of chemical ligation as Ashley et al. predicted^60^.

For the ligation of substrates with primary OH termini, T8a/T8b-p was much faster than that of D16a-p/D16b (Fig. 2c). Those containing a secondary OH group had the same tendency with l-*a*TNA more reactive than DNA. These results indicate that a mechanism in addition to the reactivity of the OH enhances l-*a*TNA ligation. The energy minimized structure of the l-*a*TNA duplex obtained from a computer calculation suggested that there is formation of a hydrogen bond between the carbonyl oxygen and the terminal OH group (Supplementary Fig. 7a–e), this would enhance nucleophilicity of the oxygen of the OH group. Another possibility is that Mn^2+^ complexes with the carbonyl of the amide, the phosphate, and the OH group to optimally position the phosphate and OH for ligation (Supplementary Fig. 7f).

### Homo- and heteroligations of DNA and l-*a*TNA are mediated by templates

**Fig. 3:**
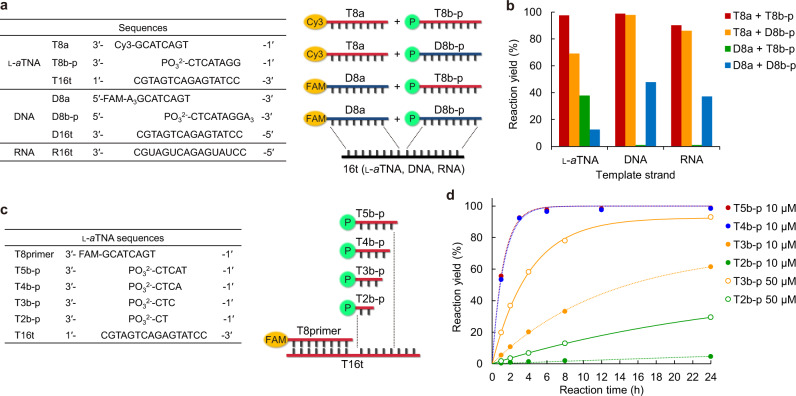
Homo- and heteroligations and template-directed incorporation of short fragment. **a** Sequences of 8-mer fragments and 16-mer template. **b** Ligation yields of indicated pairs of fragments on different templates. T8a/T8b-p (red bars), T8a/D8b-p (yellow bars), D8a/T8b-p (green bars), and D8a/D8b-p (blue bars) were ligated. Conditions: [X8a] = 0.9 μM, [X8b-p] = 1.1 μM, [X16t] = 1.0 μM, [NaCl] = 100 mM, [MnCl_2_] = 20 mM, [CNIm] = 20 mM, 4 °C, 24 h. Source data are provided as a Source Data file. **c** Sequences of 8-mer primer and fragments and 16-mer template. **d** Ligation yields for fragments of different lengths as a function of time. 5-mer fragment (red circles), 4-mer fragment (blue circles), 3-mer fragment (yellow circles), and 2-mer fragment (green circles) were used. Conditions: [T8primer] = 0.9 μM, [T16t] = 1.0 μM, [NaCl] = 100 mM, [MnCl_2_] = 20 mM, [CNIm] = 20 mM, concentration of Tnb-p strands is indicated in legend, 4 °C. Source data are provided as a Source Data file.

In order to determine whether interconversion of sequence information between l-*a*TNA and DNA or RNA is possible, ligation efficiencies of combinations of l-*a*TNA, DNA, and RNA fragments and templates were evaluated (Fig. 3a). Ligation yields after 24 h reactions indicated that T8a was more reactive than D8a (Fig. 3b and Supplementary Fig. 8), expected as the 1′-OH of l-*a*TNA has higher nucleophilicity than does the 3′-OH of DNA. Almost complete ligation of T8a/T8b-p was observed irrespective of the templates (Fig. 3b), indicating the possibility of transfer of sequence information encoded in DNA and RNA into l-*a*TNA. D8a/D8b-p ligation occurred on the l-*a*TNA template (T16t) although the yield was not high (Fig. 3b). Thus, this chemical ligation technique possibly enables interconversion of genetic information between DNA and l-*a*TNA. Ligation of fragments of different scaffolds was also observed. T8a/D8b-p was ligated in high yield on D16t and R16t templated and with slightly lower yield on T16t, whereas ligation of D8a/T8b-p was observed only on T16t (Fig. 3b). The ligation on D16t and on R16t gave comparable efficiency, presumably indicating that the helical structure of the duplexes did not so much affect the efficacy of ligation.

### l-*a*TNA ligation with short fragments

We reasoned that the high reaction rate and yield of l-*a*TNA ligation should enable the elongation of shorter fragments in a template-directed fashion. Ligation of l-*a*TNA fragments of 2-, 3-, 4-, and 5-mer (T2b-p, T3b-p, T4b-p, and T5b-p, respectively) to the 8-mer primer strand (T8primer) on T16t template was evaluated (Fig. 3c). T5b-p and T4b-p were almost completely ligated to the primer within 6 h at 4 °C with almost equivalent reaction rates of 0.82 h^−1^ for T5b-p and 0.78 h^−1^ for T4b-p (Fig. 3d and Supplementary Fig. 9). This indicates that rate determining step is the ligation and not hybridization when sufficiently long fragments are used. In contrast, about 60% of T3b-p was ligated after 24 h and the observed reaction rate was 0.056 h^−1^, and only about 10% of T2b-p was ligated under these conditions (*k*_obs_ = 0.0034 h^−1^; Fig. 3d and Supplementary Fig. 9). By increasing the concentration of fragments to 50 μM, the reaction efficacy was considerably higher; about 80% ligation of T3b-p and about 20% ligation of T2b-p was observed after 24 h (*k*_obs_ = 0.22 h^−1^ for T3b-p and *k*_obs_ = 0.018 h^−1^ for T2b-p; Fig. 3d and Supplementary Fig. 9). This suggests that the rate determining step for trimer and dimer fragments is binding to the template. Undesired intrastrand cyclization of fragments between phosphate and OH would be chemically possible^58,61^, however l-*a*TNA fragments did not show such cyclization under the present ligation conditions (Supplementary Fig. 10). These data indicated that enzyme-free primer extension of trimer fragments should be possible.

### l-*a*TNA trimers can be sequentially ligated

**Fig. 4:**
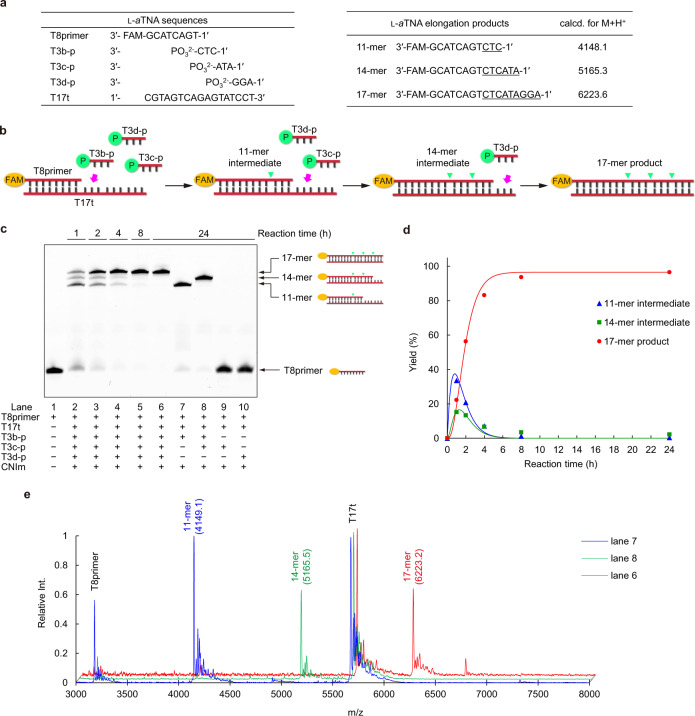
Sequential, template-directed ligation of trimers. **a** Sequences used for sequential ligation of trimers. **b** Schematic illustration of the sequential, template-directed ligation of trimers. **c** Denaturing PAGE analysis of sequential ligation. Lane 1, T8primer as a marker; lane 2, reaction products after 1 h; lane 3, reaction products after 2 h; lane 4, reaction products after 4 h; lane 5, reaction products after 8 h; lane 6, reaction products after 24 h; lane 7, reaction products in the absence of T3c-p and T3d-p; lane 8, reaction products in the absence of T3d-p; lane 9, reaction products in the absence of T3b-p and T3d-p; and lane 10, reaction products in the absence of T3b-p and T3c-p. Conditions: [T8primer] = 0.9 μM, [T3b-p] = [T3c-p] = [T3d-p] = 50 μM, [T17t] = 1.0 μM, [NaCl] = 100 mM, [MnCl_2_] = 20 mM, [CNIm] = 20 mM, 4 °C. Source data are provided as a Source Data file. **d** Ligation yield determined from PAGE. Blue triangle, green square, and red circle represent 11-mer intermediate, 14-mer intermediate, and 17-mer full-length product, respectively. Source data are provided as a Source Data file. **e** MALDI-TOF MS spectra and observed *m*/*z* of reaction mixture containing all components analyzed after 24 h (red), of reaction products in the absence of T3c-p and T3d-p, which yielded only 11-mer (blue line), and of reaction products in the absence of T3d-p, which yielded 14-mer (green line). Peaks corresponding to the template (T17t) and T8primer were also observed. Source data are provided as a Source Data file.

We first sought to demonstrate sequential ligation using the 8-mer primer (T8primer) and three trimer fragments (T3b-p, T3c-p, and T3d-p), all of which are complementary to a 17-mer template (T17t) (Fig. 4a, b). In the presence of all three trimers, the signal due to T8primer as resolved by PAGE gradually decreased and three new bands appeared that migrated more slowly (Fig. 4c), indicating elongation of T8primer. From the comparison to products of the reactions in the absence of both T3c-p and T3d-p and in the absence of T3d-p, the product bands were assigned to 11-mer and 14-mer intermediates and full-length 17-mer product. The yield of full-length product increased with time (Fig. 4d). Initially, 11- and 14-mer intermediates accumulated, then levels of the intermediates were reduced, and concurrently full-length product signal increased. After 8 h, almost all the intermediates and T8primer were converted into full-length 17-mer product. Ligation did not proceed at all without T3b-p which hybridizes to the template immediately adjacent to the primer, this demonstrated the template-directed specificity of the reaction.

Interestingly, the reaction rate in the presence of all three fragments was much faster than that of the single ligation of T3b-p under the same conditions; the single ligation of T3b-p gave about 40% conversion of the primer after 2 h, and the reaction was not completed even after 24 h. In contrast, the conversion of the primer in the presence of T3b-p, T3c-p, and T3d-p was more than 90% after 2 h and nearly quantitative production of the full-length product was observed after 24 h (Supplementary Fig. 11). The *k*_obs_ clearly reflected the difference in the efficacy: *k*_obs_ was 1.2 h^−1^ in the presence of all three fragments, whereas *k*_obs_ for ligation of the single trimer was 0.27 h^−1^. The trimers that hybridized downstream sequences presumably acted as helper strands as previously described^8,62,63^. In our case, trimer fragments would mutually assist hybridization with the template via terminal stacking interactions resulting in acceleration of the ligation through preorganized complex formation (Supplementary Fig. 12). MALDI-TOF mass spectrometry confirmed that the desired 17-mer product was successfully generated in the presence of all components; 14-mer and 11-mer intermediates were also detected (Fig. 4e). Tetramer fragments were also ligated to the primer in a sequential, template-directed manner with higher reaction rate and yield than observed with trimers (Supplementary Fig. 13). It is worth noting that almost no impurities derived from nontemplated reactions were detected by either PAGE or mass spectroscopy, indicating that complementary strand production occurs with high fidelity.

### Template-mediated synthesis of complementary l-*a*TNA strand from random trimer pool

**Fig. 5:**
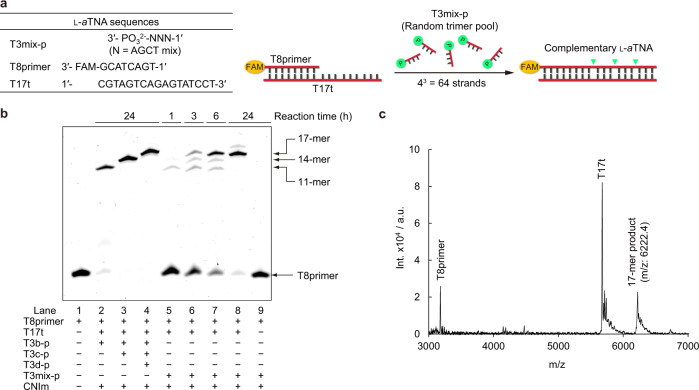
Template-directed, polymerase-like primer extension from random trimers. **a** Illustration of primer extension in the presence of T3mix-p pool. **b** Denaturing PAGE analysis of reaction with randomized trimers. Lane 1, T8primer as a marker; lane 2, reaction with only T3b-p as substrate to yield 11-mer intermediate; lane 3, reaction with T3b-p and T3c-p as substrates to yield 14-mer intermediate; lane 4, reaction with T3b-p, T3c-p, and T3d-p as substrates to yield full-length 17-mer; lane 5, reaction with T3mix-p as substrates after 1 h; lane 6, reaction with T3mix-p as substrates after 3 h; lane 7, reaction with T3mix-p as substrates after 6 h; lane 8, reaction with T3mix-p as substrates after 24 h; and lane 9, control without template. Conditions: [T8primer] = 0.9 μM, [T3mix-p] = 100 μM (total), [T17t] = 1.0 μM, [NaCl] = 100 mM, [MnCl_2_] = 20 mM, [CNIm] = 20 mM, 4 °C. Source data are provided as a Source Data file. **c** MALDI-TOF MS of reaction with T3mix-p as substrates after 24 h. Source data are provided as a Source Data file.

Finally, we demonstrate template-mediated synthesis of l-*a*TNA from random trimer pool (T3mix-p) that was chemically synthesized from l-*a*TNA phosphoramidites of four nucleobases. The extension reaction of the T8primer on the T17t template was performed in the presence of the T3mix-p (Fig. 5a). As expected, elongation of random trimer fragments into 17-mer full-length product was observed on denaturing PAGE: intermediates were produced at early stage of the reaction, whereas full-length product was observed after the consumption of intermediates (Fig. 5b). More than 75% of T8primer was converted to 17-mer full-length product after 24 h. Although a low intensity band (less than 10%) derived from unspecific ligation between template and trimer fragment was observed at the upper position, sufficiently selective elongation reaction was confirmed. MALDI-TOF mass spectrometry of the reaction mixture indicated that elongation resulted in an oligomer of sequence complementary to the template (Fig. 5c). Neither intermediates nor full-length product were observed in the absence of the template T17t. These results clearly demonstrated that template-directed polymerization of l-*a*TNA was achieved nonenzymatically utilizing a simple chemical catalyst without any special modification on the polymerized trimers. To the best of our knowledge, this is the first example of template-directed polymerase-like, chemical primer extension of acyclic XNA from a random fragment pool. Importantly, the natural phosphodiester linkage was generated without any special modification of the l-*a*TNA fragments through a fast and effective chemical ligation.

The template-directed, chemical ligation of l-*a*TNA using CNIm was considerably faster than ligation of DNA under the same conditions. Homo- and heteroligations of l-*a*TNA and DNA were also observed. This indicates that genetic information encoded in DNA and RNA sequence could possibly be transcribed into l-*a*TNA, and also reverse transcribed into DNA. The l-*a*TNA ligation was also observed when dimers were used as substrates although the reaction was less efficient than was ligation of longer fragments. Sequential ligation of l-*a*TNA with three trimer fragments was accomplished, and almost complete conversion into full-length product was attained. No products noncomplementary to the template were detected. The selective, template-directed elongation of l-*a*TNA was demonstrated in the presence of a random l-*a*TNA trimer pool, establishing that synthesis of a complementary strand was possible. To the best of our knowledge, this is the first report of template-directed synthesis of an acyclic XNA containing all four nucleobases by generating phosphodiester linkages without enzymes.

High reaction rate and yield was observed in the presence of condensation reagent but without any special modification on the fragments. As XNAs have acyclic scaffolds very different structurally from natural nucleic acid, it might be difficult to engineer a polymerase for primer extension of XNA. Our strategy for enzyme-free copying of sequence information of acyclic XNA is an attractive system for replication for construction of artificial life^64–66^ and highly functional biological tools composed of acyclic XNA via in vitro selection^67,68^.

Our data indicate that l-*a*TNA could have been a precursor of RNA, because the ligation of l-*a*TNA on DNA and RNA templates and of DNA on an l-*a*TNA template was possible using CNIm, a possible prebiotic condensation agent^69^. l-threoninol, a scaffold of l-*a*TNA, could be generated by reduction of l-threonine or its precursor that was possibly generated by prebiotic chemistry^70^. Possibility of prebiotic synthesis of nucleobase-acetic acid has already been shown as potential component of PNA^71^. Therefore, l-*a*TNA has a potential to be synthesized at prebiotic conditions. Our results show that exchange of sequence information between l-*a*TNA and the nucleic acids of the modern world is possible. The nonenzymatic, template-directed l-*a*TNA elongation is the foundation for a new genetic system of the XNA world.

## Methods

All experimental procedures and characterization data are provided in Supplementary Information.

### General procedure for chemical ligation by CNIm

A typical reaction in 30 μL volume was performed as follows: an aliquot of solution containing the required amount of oligomer was added to 6.0 μL of 500 mM NaCl aq and 3.0 μL of 200 mM MnCl_2_ aq. The mixture was diluted with autoclaved Milli-Q water to 27 μL before starting the reaction by the addition of 3.0 μL 200 mM *N*-cyanoimidazole aq. The desired temperature was maintained using a thermal cycler. For the time-course evaluation, 4.0 μL of the reaction mixture was taken into another microtube, and 4.0 μL of quenching mix (aqueous solution of 12.5 mM NaOH and 125 mM EDTA) was added. After the addition of 2.0 μL loading buffer (glycerol:formamide:0.5 M EDTA, 6:6:1v/v/v, containing 0.06 % bromophenol blue), 2.5 μL of the quenched sample was applied to a denaturing PAGE (20% acrylamide, 8 M urea). Electrophoresis was performed at 4 W for 2–3 h. We found that denaturing PAGE does not completely dissociate an l-*a*TNA/l-*a*TNA duplex when the sequence is long, due to high duplex stability. We therefore kept the temperature of electrophoresis apparatus below room temperature so that only unreacted 8-mer primer dissociated from the template. Under these PAGE conditions, elongation products of longer than 11-mer formed duplexes with the template during PAGE, resulting in a large difference in migration distance between the primer and the products. After the run, gel was analyzed using a Typhoon FLA 9500, and intensities of gel bands were quantified using an ImageQuant TL. *k*_obs_ was estimated by data fitting assuming first-order reactions, using yield in the first three time points at time-course experiment.

### MALDI-TOF MS analysis of reaction mixtures

A 50-μL aliquot of the reaction mixture was added to 7.0 μL of 3.0 M sodium acetate aq and 120 μL of absolute ethanol, and the solution was mixed well. After centrifuging at 16,000 × *g* for 5 min at 4.0 °C, 120 μL aqueous solution of 70% (v/v) ethanol was added and mixed before centrifuging at 16,000 × *g* for 5 min at 4.0 °C. The supernatant was decanted, and the pellet was air-dried. The residue was dissolved in 5.0 μL of water and was analyzed by MALDI-MS using 3-hydroxypicolinic acid as a matrix.

### Statistics and reproducibility

Experiments in Figs. 1d, e, 4c, and 5b and Supplementary Fig. 6 were repeated three times and experiments in Supplementary Figs. 2–4, 8, 9, 11a, and 13c were repeated twice, which gave independently similar results.

### Reporting summary

Further information on research design is available in the Nature Research Reporting Summary linked to this article.

## Supplementary information

Supplementary Information

Reporting Summary

## Data Availability

All data presented in the manuscript are available upon request from the corresponding authors. Raw data are provided as a Source Data file. Source data are provided with this paper.

## Source data

Source Data
