# Supplementary material for: Nonenzymatic polymerase-like template-directed synthesis of acyclic l-threoninol nucleic acid
Source: Nat Commun. 2021 Feb 5;12:804. doi: 10.1038/s41467-021-21128-0 (PMC7864931; doi:10.1038/s41467-021-21128-0)
Supplement: Supplementary file 1 — Supplementary Information [file 41467_2021_21128_MOESM1_ESM.pdf]

Supplementary Information

Non-enzymatic polymerase-like template-directed synthesis of acyclic L-threoninol nucleic acid

Keiji Murayama, Hikari Okita, Takumi Kuriki, and Hiroyuki Asanuma

Graduate School of Engineering, Nagoya University,
Furo-cho, Chikusa-ku, Nagoya 464-8603, Japan

Table of Contents

1. Supplementary Methods……………………………………………………………………S2
2. Supplementary Figures and Table (Supplementary Figure S1-S12, Supplementary Table S1)…S3
3. Supplementary Note (MALDI TOF-MS, HPLC charts)…………………………………S13

1. Supplementary Methods

Materials: Reagents for oligomer synthesis and Poly-Pak II cartridges were purchased from Glen Research. The column for HPLC purification was purchased from Kanto Chemical Co., Ltd. L-*a*TNA oligomers (with exceptions of T8a and T8a-p) and DNA and RNA oligonucleotides were purchased from Hokkaido System Science Co., Ltd. and were of HPLC grade. The oligomer and oligonucleotides used in the study are listed in Supplementary Table 1. *N*-Cyanoimidazole was purchased from Toronto Research Chemicals, and stored at -80 ^{o}C.

Synthesis and purification of L-*a*TNA oligomers: L-*a*TNA phosphoramidite monomers involving T, G, A, and C were synthesized following reported procedures.[1] T8a and T8a-p were synthesized using H-8-SE DNA/RNA synthesizer using phosphoramidite chemistry. Cy3-conjugated CPG was used as a solid support.[2] For the coupling of all monomers, incubation time was extended to 600 s. The concentrations of L-*a*TNA phosphoramidite monomers were 0.1 M for G and 0.75 M for T, A, and C. Synthesized L-*a*TNAs were purified using Poly-Pak II cartridges (Glen Research) and reversed-phase HPLC (Kanto Chemical, Mightysil RP-18 GPII column and JASCO, ChromNAV). After purification, synthesized oligonucleotides were characterized by MALDI-TOF MS.

Melting-temperature measurements: Melting curves of duplexes were obtained using a Shimadzu UV-1800 (Tm Analysis Software) equipped with a programmable temperature controller by measuring the change in absorbance at 260 nm versus temperature. The temperature ramp was 0.5 ^{o}C min^{-1}. The melting temperature (T_m) was determined from the maximum in the first derivative of the melting curve by Spectra Manager (JASCO). Both the heating and cooling curves were measured, and the T_m measurements obtained agreed within 2.0 ^{o}C.

Computer simulation of structures of L-*a*TNA duplex: Maestro Version 10.7 (Schrödinger) was used to obtain optimized geometries and energy minimized structures. Torsional sampling (MCMM) was employed for the calculation. After 2000 search steps, each structure was minimized, and the most stable geometry was selected. The OPLS_2005 force field was used for the calculations.

2. Supporting Figures and Tables

Supplementary Table 1. All sequences of oligomer used in this study and result of characterization by MALDI-TOF MS

	Name	Sequence	Calcd. [M+H$^+$]	Obsd. MS
L-*a*TNA	T8a	3′-Cy3-GCATCAGT-1′	3177.9	3179.0
	T8a-p	3′-Cy3-GCATCAGT-PO_3^{2-}-1′	3257.9	3258.8
	T8b	3′-CTCATAGG-1′	2641.7	2640.7
	T8b-p	3′-PO_3^{2-}-CTCATAGG-1′	2721.7	2721.6
	T16t	3′-CCTATGAGACTGATGC-1′	5344.4	5345.8
	T17t	3′-TCCTATGAGACTGATGC-1′	5677.4	5678.9
	T8primer	3′-FAM-GCATCAGT-1′	3178.8	3179.1
	T5b-p	3′-PO_3^{2-}-CTCAT-1′	1663.4	1662.3
	T4b-p	3′-PO_3^{2-}-CTCA-1′	1330.3	1329.3
	T4c-p	3′-PO_3^{2-}-TAGG-1′	1410.3	1409.3
	T3b-p	3′-PO_3^{2-}-CTC-1′	988.3	987.3
	T3c-p	3′-PO_3^{2-}-ATA-1′	1036.3	1035.3
	T3d-p	3′-PO_3^{2-}-GGA-1′	1077.3	1076.3
	T2b-p	3′-PO_3^{2-}-CT-1′	670.2	N.D.
DNA	D8a	5′-FAM-A_3GCATCAGT-3′	3885.8	3886.4
	D8b-p	5′-PO_3^{2-}-CTCATAGG A_3-3′	3428.6	3429.1
	D16t	5′-CCTATGAGACTGATGC-3′	4879.9	4884.1
	D16a	5′-FAM-GTGACGTAGCATCAGT-3′	5457.1	5458.8
	D16a-p	5′-FAM-GTGACGTAGCATCAGT-PO_3^{2-}-3′	5537.0	5537.8
	D16b	5′-CTCATAGGAACTCTCG-3′	4839.9	4840.3
	D16b-p	5′-PO_3^{2-}-CTCATAGGAACTCTCG-3′	4919.9	4921.0
	D32t	5′-CGAGAGTTCCTATGAGACTGATGCTACGTCAC-3′	9820.8	9827.2
RNA	R16t	5′-CCUAUGAGACUGAUGC-3′	5080.0	5087.9

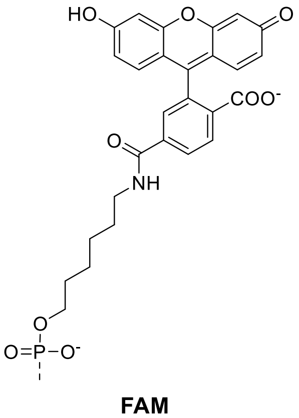

FAM

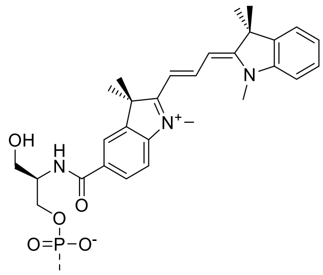

Cy3

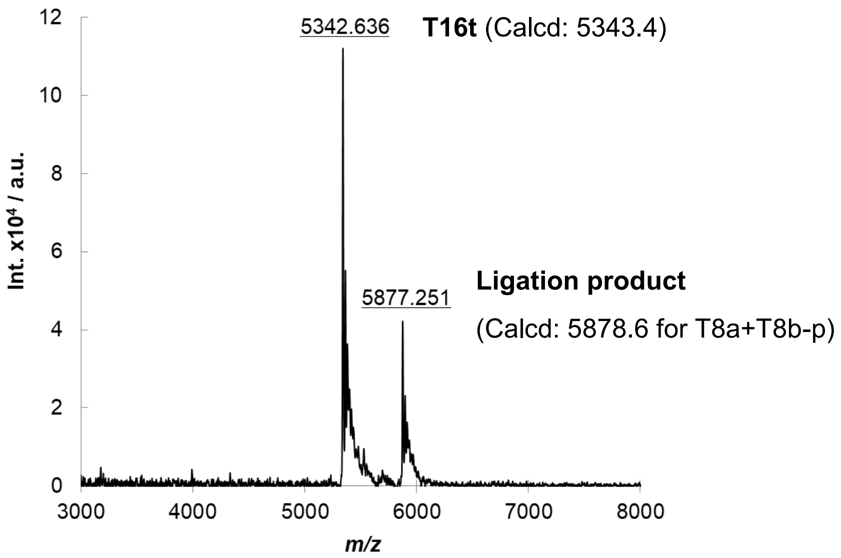

Supplementary Figure 1. MALDI-TOF MS spectrum of a reaction mixture containing T8a, T8b-p, and T16t. Calcd: 5878.6, Obs: 5877.3 for ligation product (T8a+T8b-p). Reaction conditions: [T8a] = 0.9 μM, [T8b-p] = 1.1 μM, [T16t] = 1.0 μM, [NaCl] = 100 mM, [$MnCl_2$] = 20 mM, [CNIm] = 20 mM, 4 °C for 24 h.

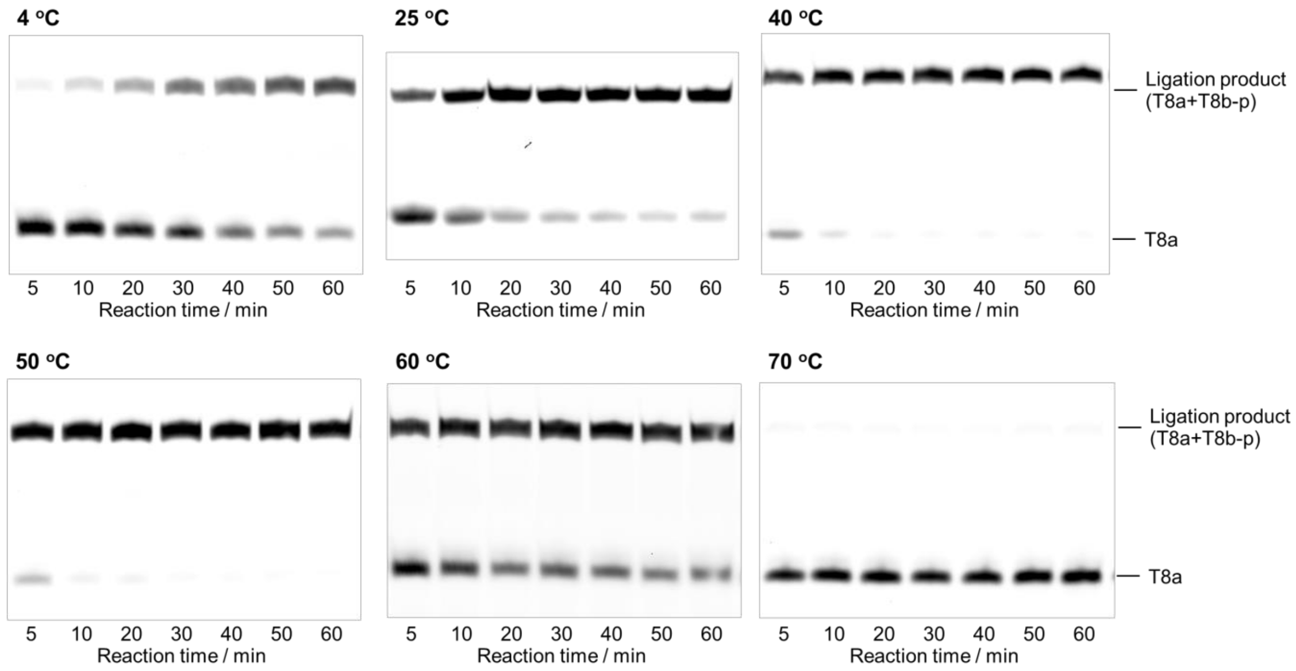

Supplementary Figure 2. Denaturing PAGE analyses of T8a and T8b-p ligation reactions on T16t as a function of time at indicated temperatures. Conditions: [oligomers] = 1.0 μM, [NaCl] = 100 mM, [$MnCl_2$] = 20 mM, [CNIm] = 20 mM.

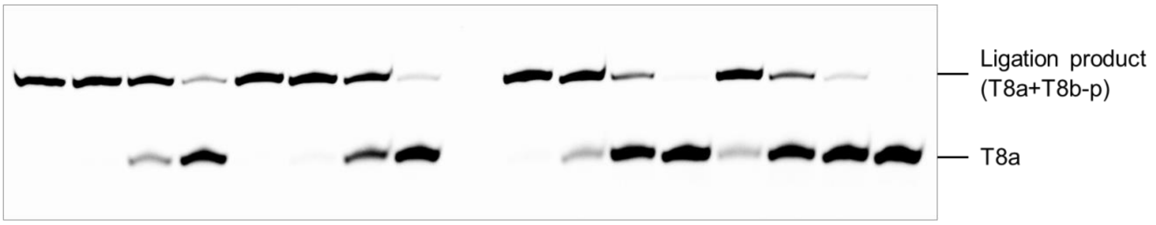

Lane	1	2	3	4	5	6	7	8	9	10	11	12	13	14	15	16
[Mn2+] / mM	100				20				5.0				1.0			
[CNIm] / mM	100	20	5.0	1.0	100	20	5.0	1.0	100	20	5.0	1.0	100	20	5.0	1.0
Yield / %	>99.9	99.6	82.5	10.9	99.8	99.0	58.6	3.5	99.4	87.4	16.3	0.6	87.9	20.0	3.6	0

Supplementary Figure 3. Denaturing PAGE analyses of T8a and T8b-p ligation reaction in the presence of indicated concentrations of CNIm and Mn^{2+}. Conditions: [T8a] = 0.9 μM, [T8b-p] = 1.1 μM, [T16t] = 1.0 μM, [NaCl] = 100 mM, [$MnCl_2$] = 1.0-100 mM, [CNIm] = 1.0-100 mM, 25 °C for 30 min.

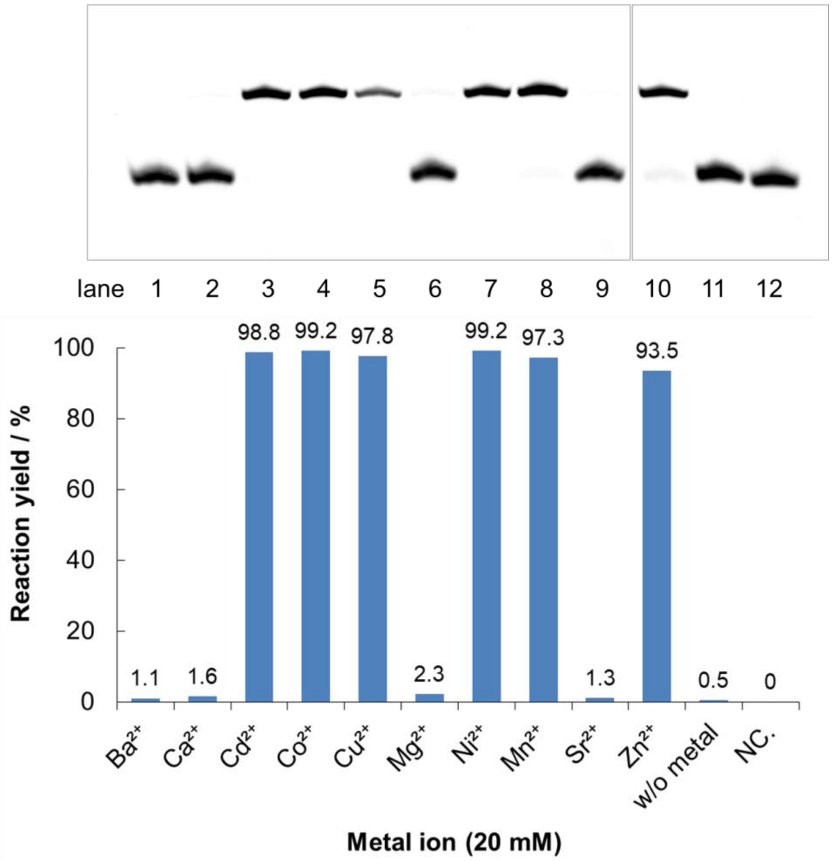

Supplementary Figure 4. Ligation yields in the presence of various divalent metal ions. Upper: Denaturing PAGE of ligation reaction of T8a and T8b-p. Lane 1, $BaCl_2$; Lane 2, $CaCl_2$; Lane 3, $CdCl_2$; Lane 4, $CoCl_2$; Lane 5, $CuCl_2$; Lane 6, $MgCl_2$; Lane 7, $NiCl_2$; Lane 8, $MnCl_2$; Lane 9, $SrCl_2$; Lane 10, $ZnCl_2$; Lane 11, control without metal ion; Lane 12, T8a as marker. Lower: Ligation yield calculated from band intensity of the gel. Conditions: [T8a] = 0.9 μM, [T8b-p] = 1.1 μM, [T16t] = 1.0 μM, [NaCl] = 100 mM, [M^{2+}] = 20 mM, [CNIm] = 20 mM, 25 °C for 30 min. Note: Total intensity of band in lane 5 was lower than in the other lanes, probably due to chemical bleaching of the fluorophore in the presence of Cu^{2+}.

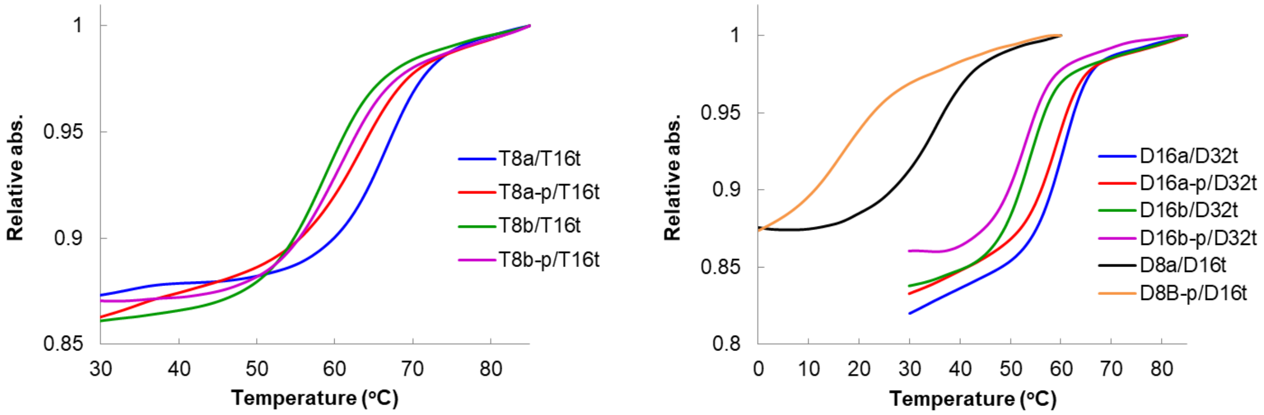

Supplementary Figure 5. Melting profiles of duplexes formed between template and fragments. Conditions: 100 mM NaCl, 10 mM phosphate buffer (pH 7.0), 2.0 μM each oligonucleotide.

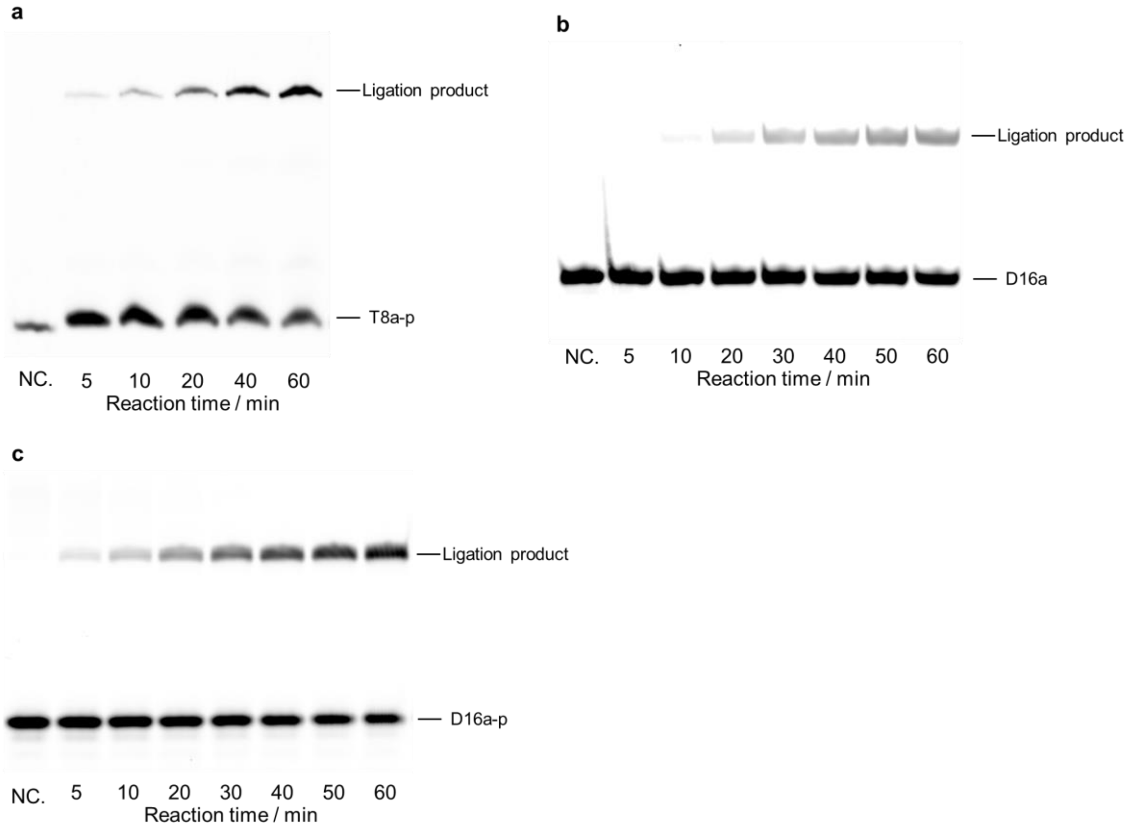

Supplementary Figure 6. Denaturing PAGE analyses of effects of position of phosphate and thermal stability on the ligation efficacy. **a**: Reaction of T8a-p and T8b in the presence of T16t. **b**: Reaction of D16a and D16b-p in the presence of D32t. **c**: Reaction of D16a-p and D16b in the presence of D32t. Conditions: [oligomers] = 1.0 μM, [NaCl] = 100 mM, [$MnCl_2$] = 20 mM, [CNIm] = 20 mM, 25 °C.

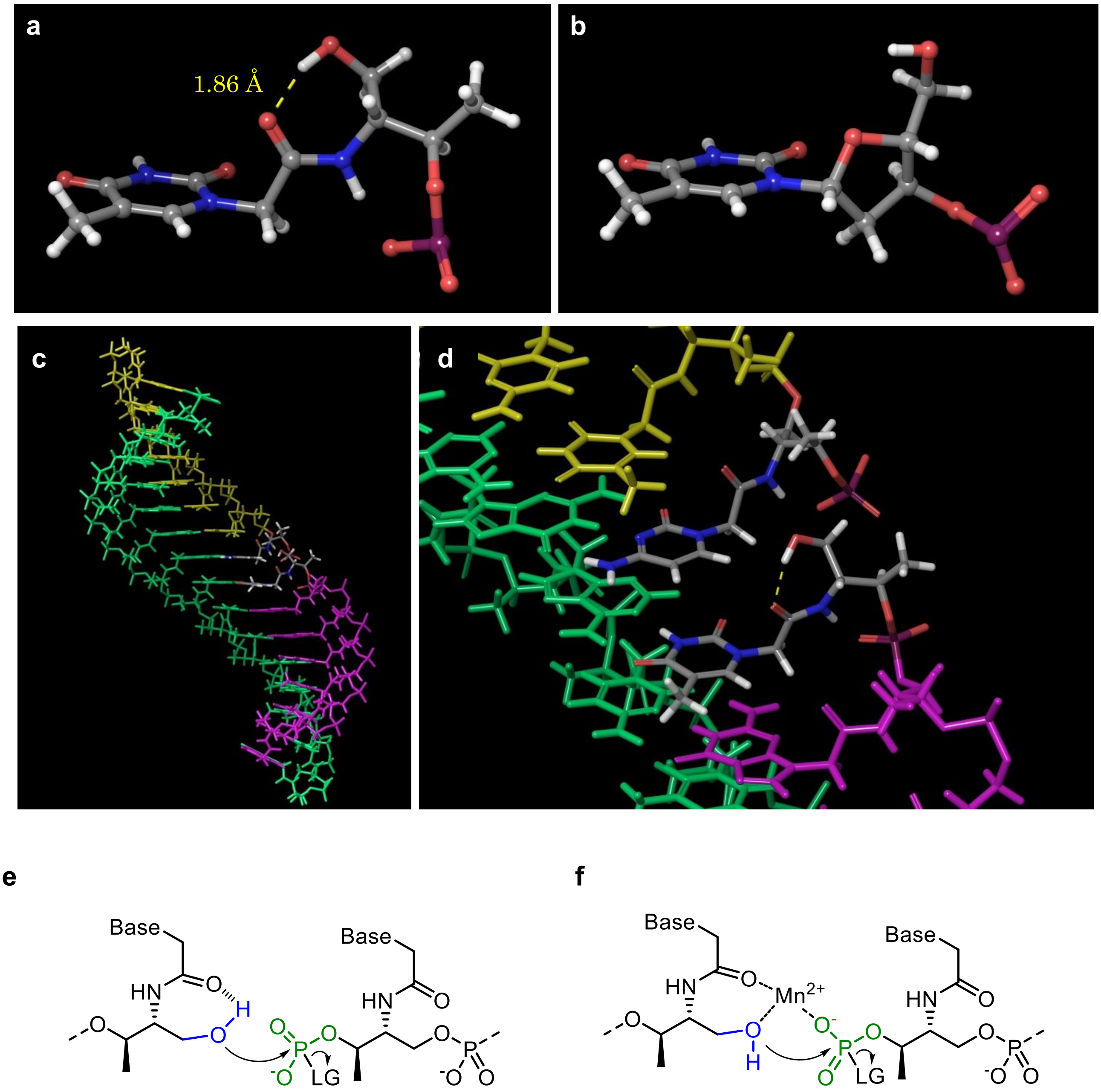

Supplementary Figure 7. Structural analysis for discussion on efficiency of chemical ligation. **a-b,** Energy minimized structure of a) L-*a*TNA thymine monomer with primary 1'-OH and b) DNA thymine monomer with primary 5'-OH. **c-d,** Energy minimized structure of nicked L-*a*TNA duplex partially based on the minimized structure of L-*a*TNA monomer. Green strand, purple strand, and yellow strand shows T16t, T8a, and T8b-p, respectively. **e**, Hydrogen bonding between oxygen in carbonyl and hydrogen in hydroxyl group may enhance nucleophilicity of oxygen of the hydroxyl group. **f**, The oxygen in the carbonyl may be a ligand of Mn^{2+}, which could optimally position the hydroxyl group and the phosphate group.

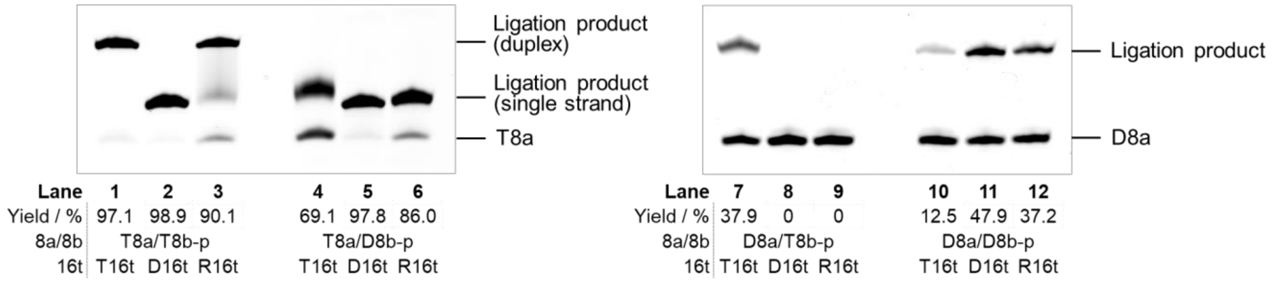

Supplementary Figure 8. Denaturing PAGE analyses of homo- and hetero-ligations on L-*a*TNA, DNA, and RNA templates. Conditions: [X8a] = 0.9 μM, [X8b-p] = 1.1 μM, [X16t] = 1.0 μM, [NaCl] = 100 mM, [$MnCl_2$] = 20 mM, [CNIm] = 20 mM, 4 °C for 24 h.

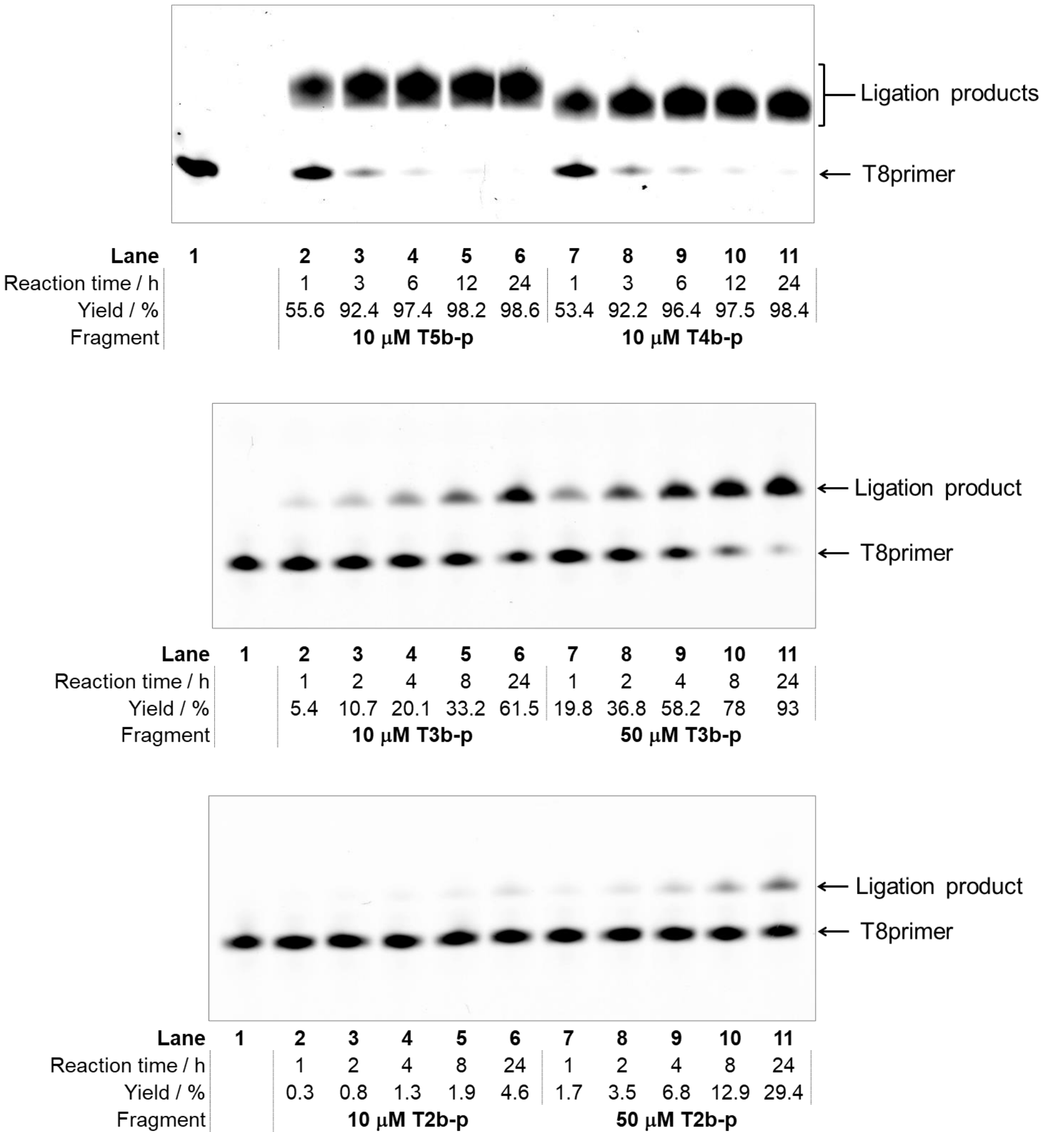

Supplementary Figure 9. Denaturing PAGE analyses of chemically facilitated, template-mediated ligation of L-*a*TNA primer and indicated fragments. Conditions: [T8primer] = 0.9 μM, [T16t] = 1.0 μM, [NaCl] = 100 mM, [$MnCl_2$] = 20 mM, [CNIm] = 20 mM, 4 °C for indicted times. Running at 50 °C for complete dissociation of duplexes between template and elongated product. The concentrations of Tnb-p strands are indicated. Lane 1 contained only T8primer.

2-mer L-*a*TNA fragment (3’-PO_3^{2-}-CT-1’)

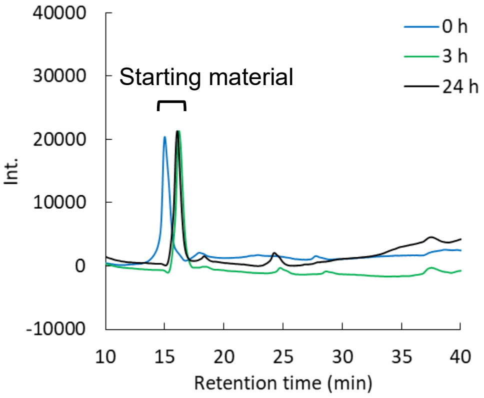

3-mer L-*a*TNA fragment (3’-PO_3^{2-}-ACT-1’)

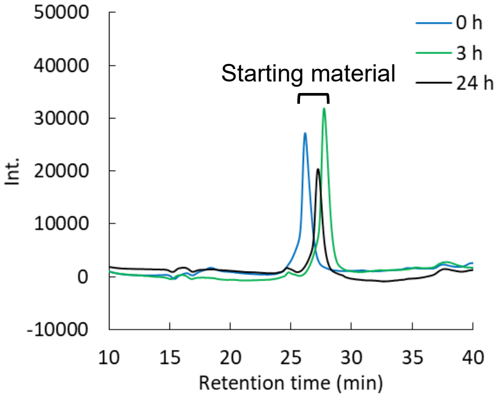

4-mer L-*a*TNA fragment (3’-PO_3^{2-}-CTCG-1’)

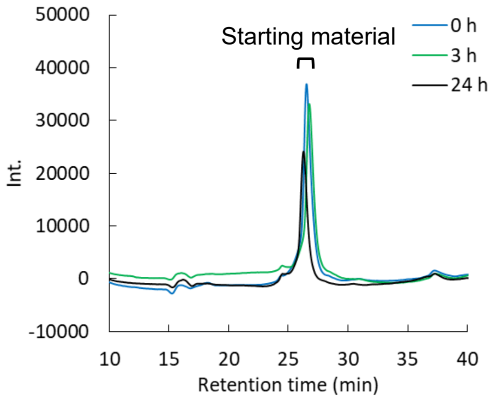

Result of MALDI-TOF MS (m/z)

	Reaction time		
	0 h	3 h	24 h
2-mer (Calcd. 669.2)	N.D.	N.D.	N.D.
3-mer (Calcd. 1011.3)	1011.5	1012.8	1012.4
4-mer (Calcd. 1345.3)	1346.6	1346.8	1346.4

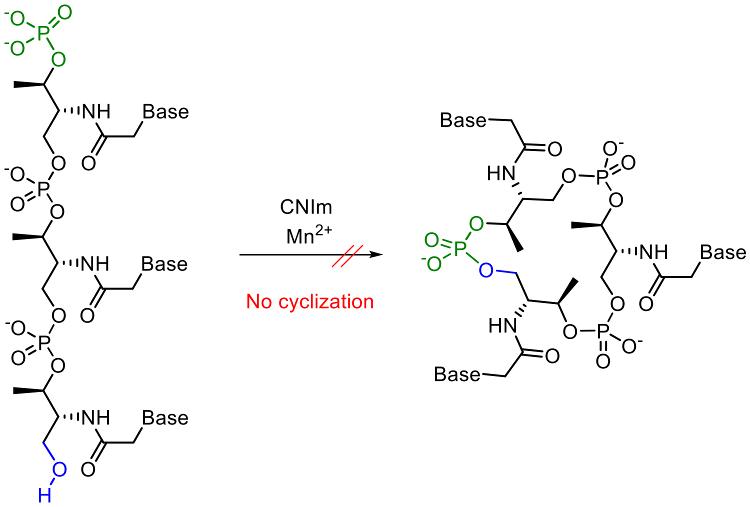

Supplementary Figure 10. HPLC analyses of intrastrand ligation of indicated L-*a*TNA fragments. Conditions: [fragment] = 300 μM, [NaCl] = 100 mM, [$MnCl_2$] = 20 mM, [CNIm] = 20 mM, 4 °C for indicted times. After quenching of the reaction, the reaction mixture was neutralized by HCl aq before injection into HPLC. From 0% buffer B to 5% buffer B (Buffer A: 50 mM ammonium formate, Buffer B: mixture of 50 mM ammonium formate and acetonitrile (50:50, v/v)). Corrected fraction was characterized by MALDI-TOF. Bottom scheme shows that cyclization of L-*a*TNA fragment was not available.

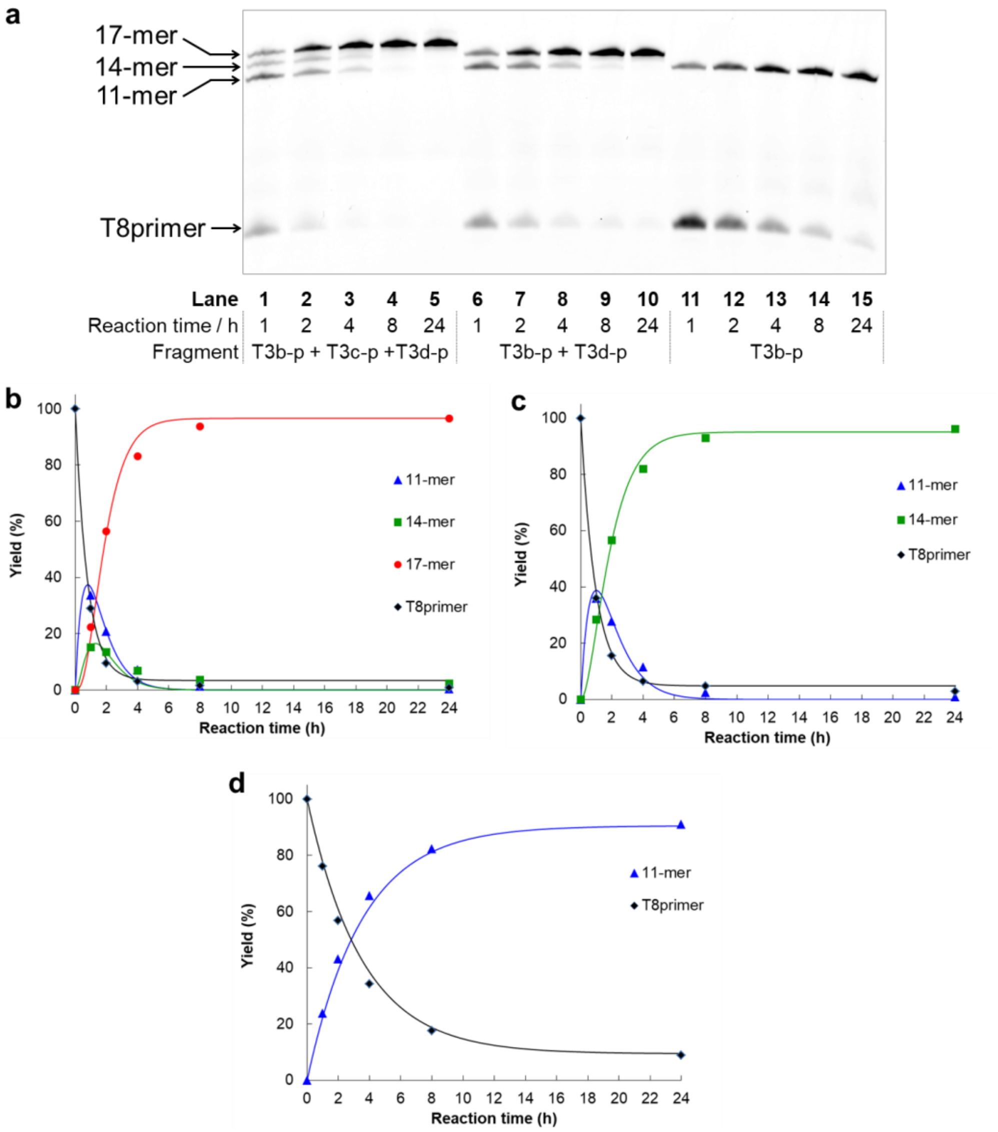

Supplementary Figure 11. Ligation yields in the presence of indicated trimer fragments for indicated reaction times**. a,** Denaturing PAGE analyses of reactions. **b-d,** Primer amount and products formed over time in the presence of b) T3b-p, T3c-p, and T3d-p; c) T3b-p and T3c-p; and d) T3b-p. Conditions: [T8primer] = 0.9 μM, [T3b-p] = [T3c-p] = [T3d-p] = 50 μM, [T17t] = 1.0 μM, [NaCl] = 100 mM, [$MnCl_2$] = 20 mM, [CNIm] = 20 mM, 4 °C. We analyzed the ligation reaction in the absence of T8primer by MALDI-TOF and found no ligation product (data not shown).

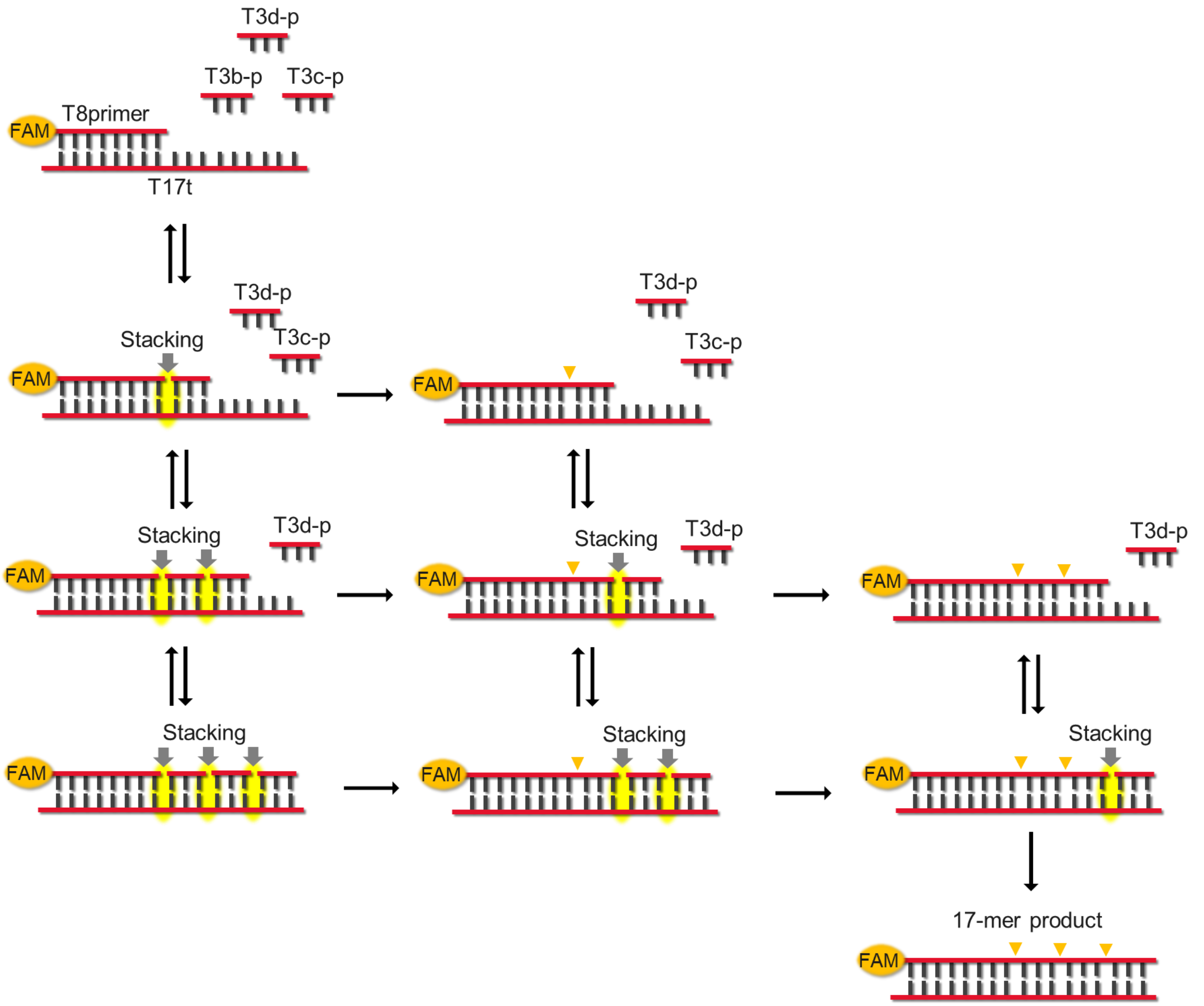

Supplementary Figure 12. Predicted mechanism of acceleration of the ligation reaction by short downstream fragments. Downstream trimer fragments would mutually assist hybridization with the template via terminal stacking interactions even before ligation. As a result, the ligation reaction could accelerate through pre-organized complex formation.

a

	L-*a*TNA sequences
T8primer	3′-FAM-GCATCAGT-1′
T4b-p	3′-PO_3^{2-}-CTCA-1′
T4c-p	3′-PO_3^{2-}-TAGG-1′
T16t	1′-CGTAGTCAGAGTATCC-3′

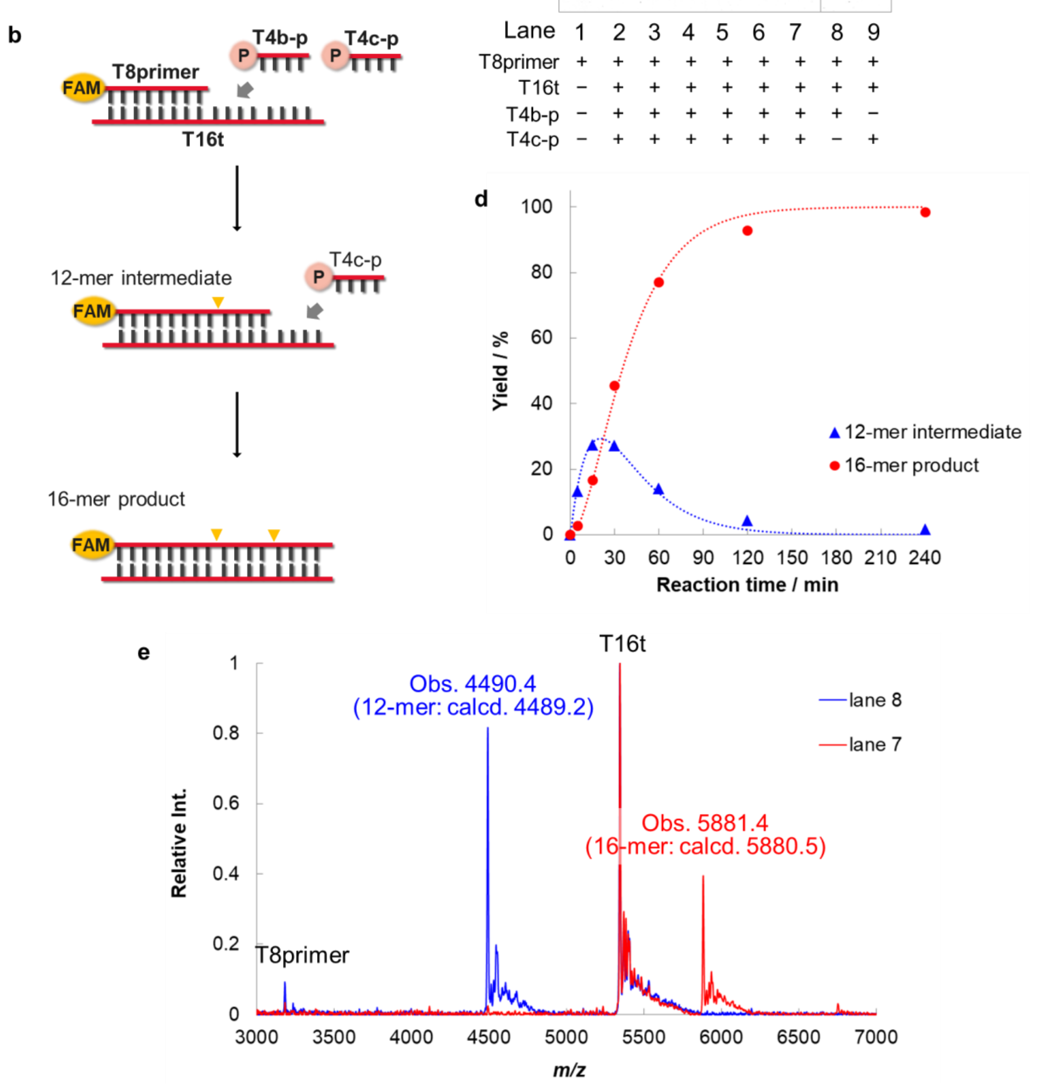

Supplementary Figure 13. Sequential ligation of tetramer fragments on the template. **a,** Sequences used for experiments with tetramer fragments. **b,** Schematic of sequential ligation of tetramer fragments. **c**, Denaturing PAGE analysis of reactions. Lane 1, T8primer as a marker; Lane 2, reaction products after 5 min; Lane 3, reaction products after 15 min; Lane 4, reaction products after 30 min; Lane 5, reaction products after 1 h; Lane 6, reaction products after 2 h; Lane 7, reaction products after 4 h; Lane 8, reaction products in the absence of T4c-p; Lane 9, reaction products in the absence of T4b-p. **d**, Plot of yield versus reaction time. **e**, MALDI-TOF MS spectra of the reaction mixtures from lane 7 (red line) and lane 8 (blue line). Conditions: [T8primer] = 0.9 μM, [T16t] = 1.0 μM, [T4b-p] = [T4c-p] = 10 μM, [NaCl] = 100 mM, [$MnCl_2$] = 20 mM, [CNIm] = 20 mM, 25 °C.

3. Supplementary Note

3-1. Results of MALDI-TOF MS for synthesized sequences:

T8a: Obsd. m/z 3179.0 (Calcd. for [T8a + H^+]: m/z 3177.9)

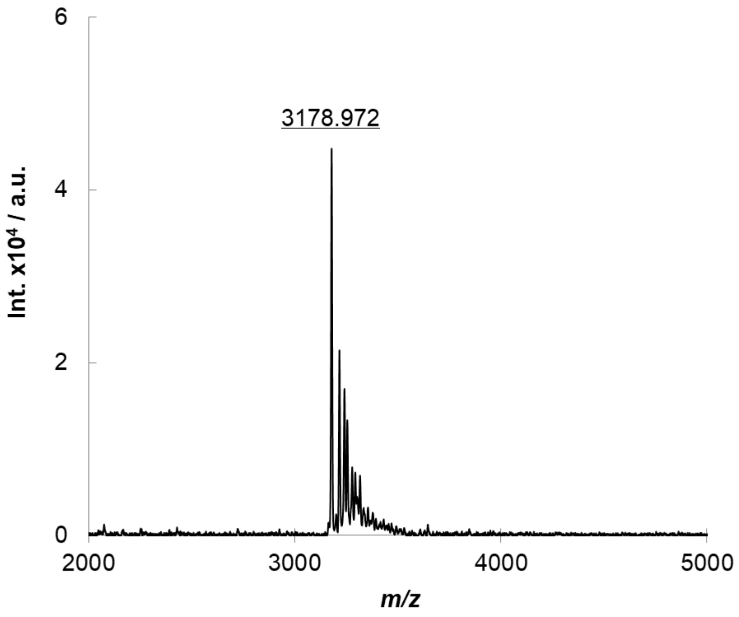

T8a-p: Obsd. m/z 3258.8 (Calcd. for [T8a-p + H^+]: m/z 3257.9)

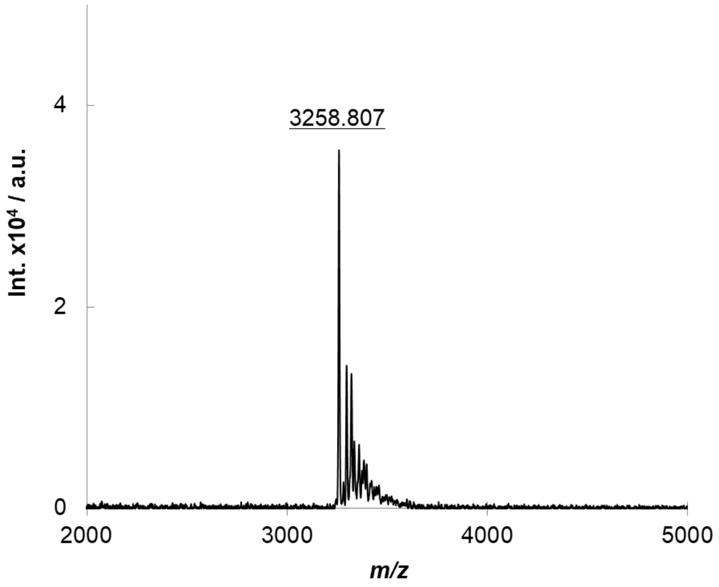

3-2. Results of HPLC for synthesized sequences:

Buffer A: 50 mM ammonium formate

Buffer B: mixture of 50 mM ammonium formate and acetonitrile (50:50, v/v)

From 35% buffer B to 55% buffer B.

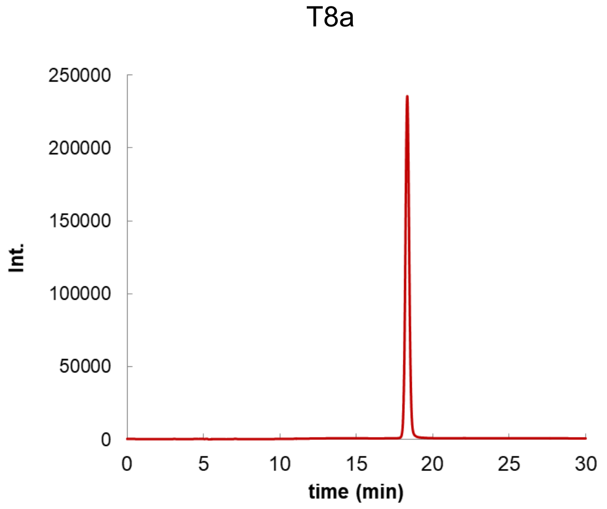

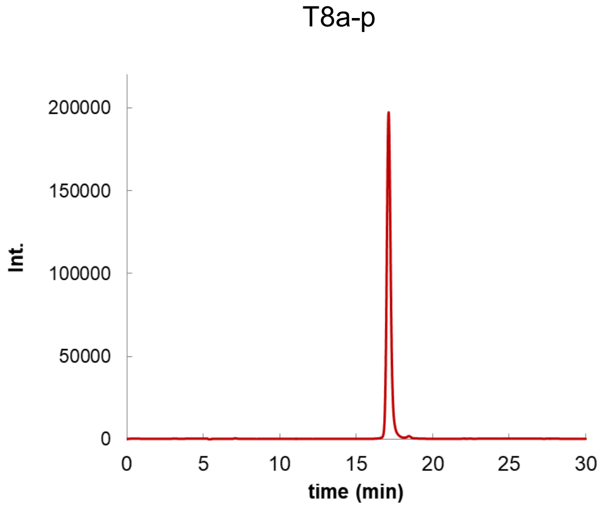

Supplementary References

(1) Murayama, K., Kashida, H., Asanuma, H. *Chem. Commun.* **2015**, *51*, 6500–6503.

(2) Murayama, K.; Kashida H.; Asanuma, H. *Method in Molecular Biology*, **2019**, Vol. 1973, Non-Natural Nucleic Acids.
